# Supplementary material for: Hollow Fiber Polyimide Membranes Prepared in a Triple Orifice Spinneret: Effect of a Reduced Water Activity in the Bore Fluid on the Gas Separation Performance
Source: Polymers (Basel). 2021 Jul 4;13(13):2211. doi: 10.3390/polym13132211 (PMC8272205; doi:10.3390/polym13132211)
Supplement: Supplementary file 1 [file polymers-13-02211-s001.zip › polymers-1266970-supplementary.pdf]

Supporting Info

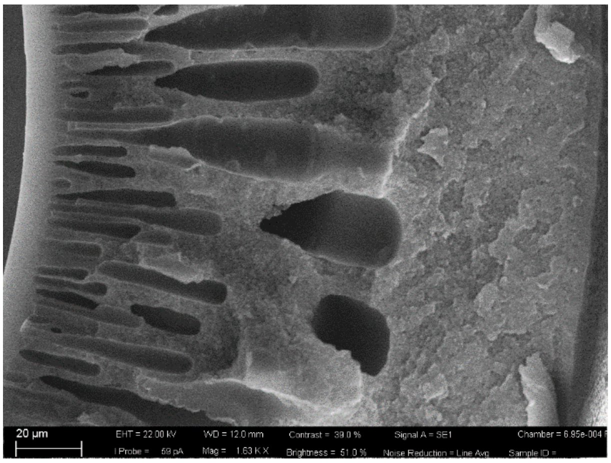

a)

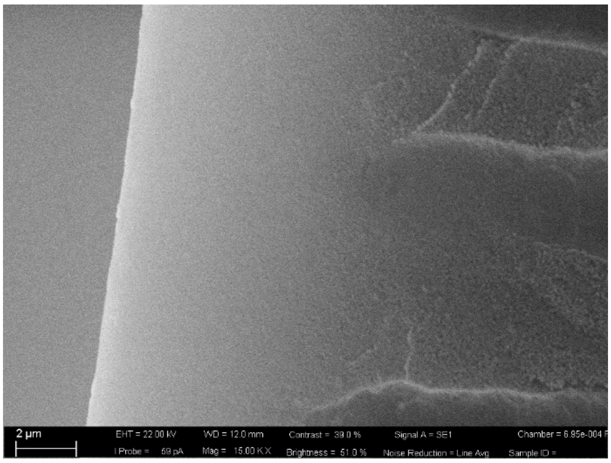

b)

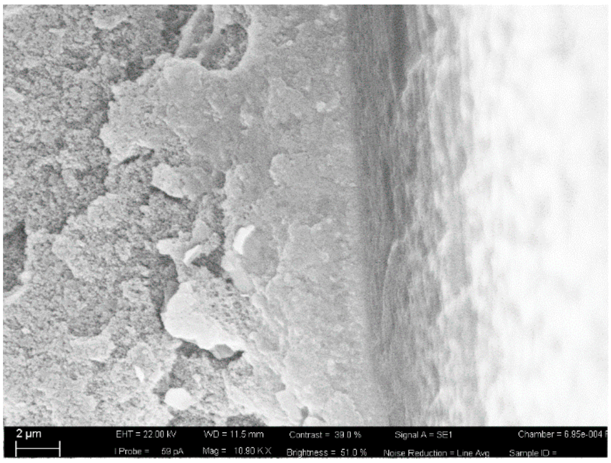

c)

H0 (BF=water; no EF)

Figure S1. SEM images of H0 HF: cross-section (a), magnification of the inner skin layer (b) and outer skin layer (c).

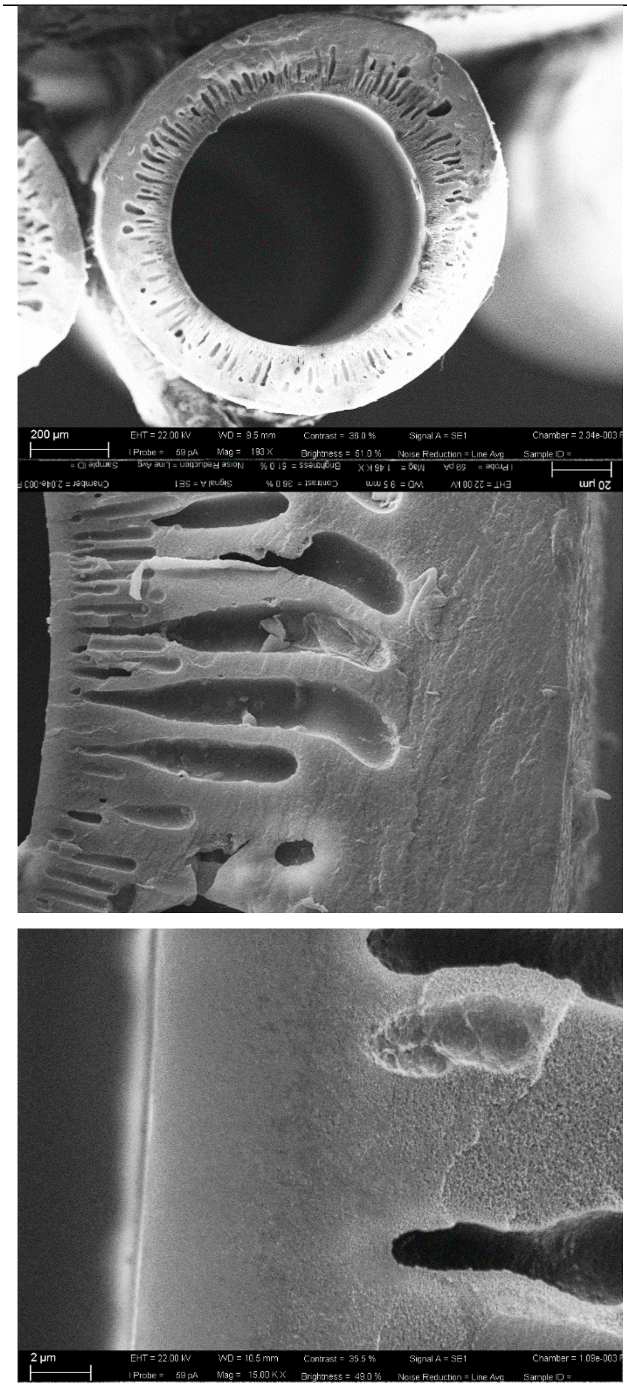

H1 (BF=water)

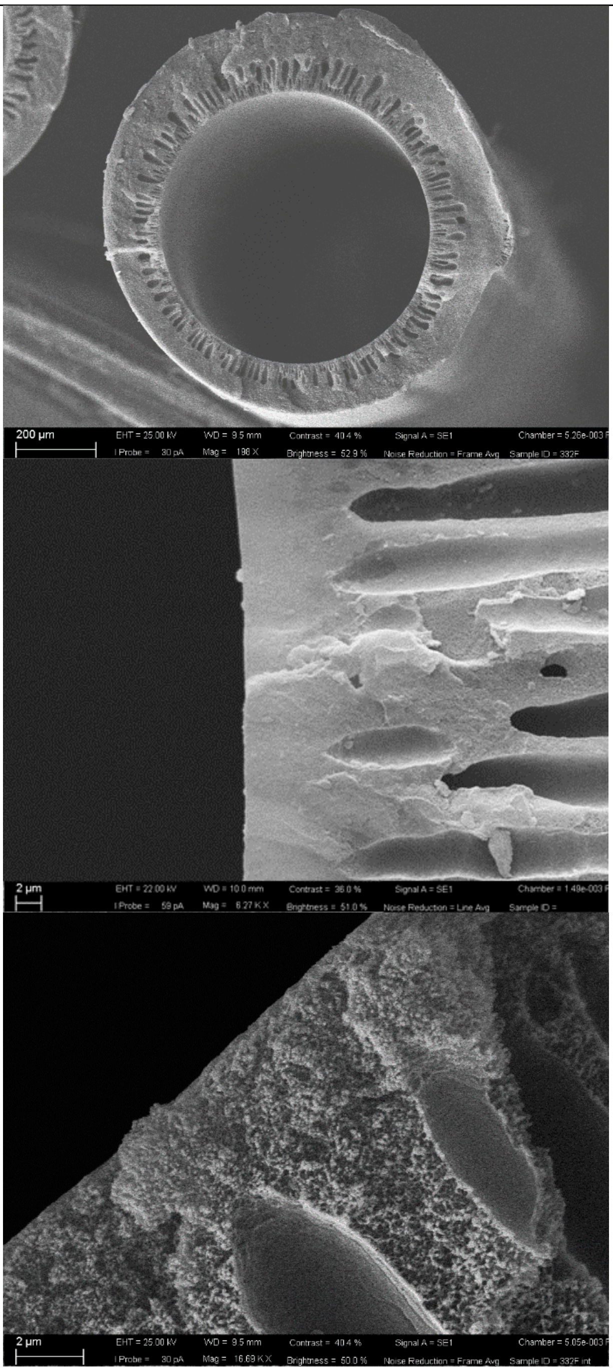

H4 (BF=NMP/water 60/40)

Figure S2. SEM images of H1 and H4 HFs: cross-section and magnification of the inner skin layer.

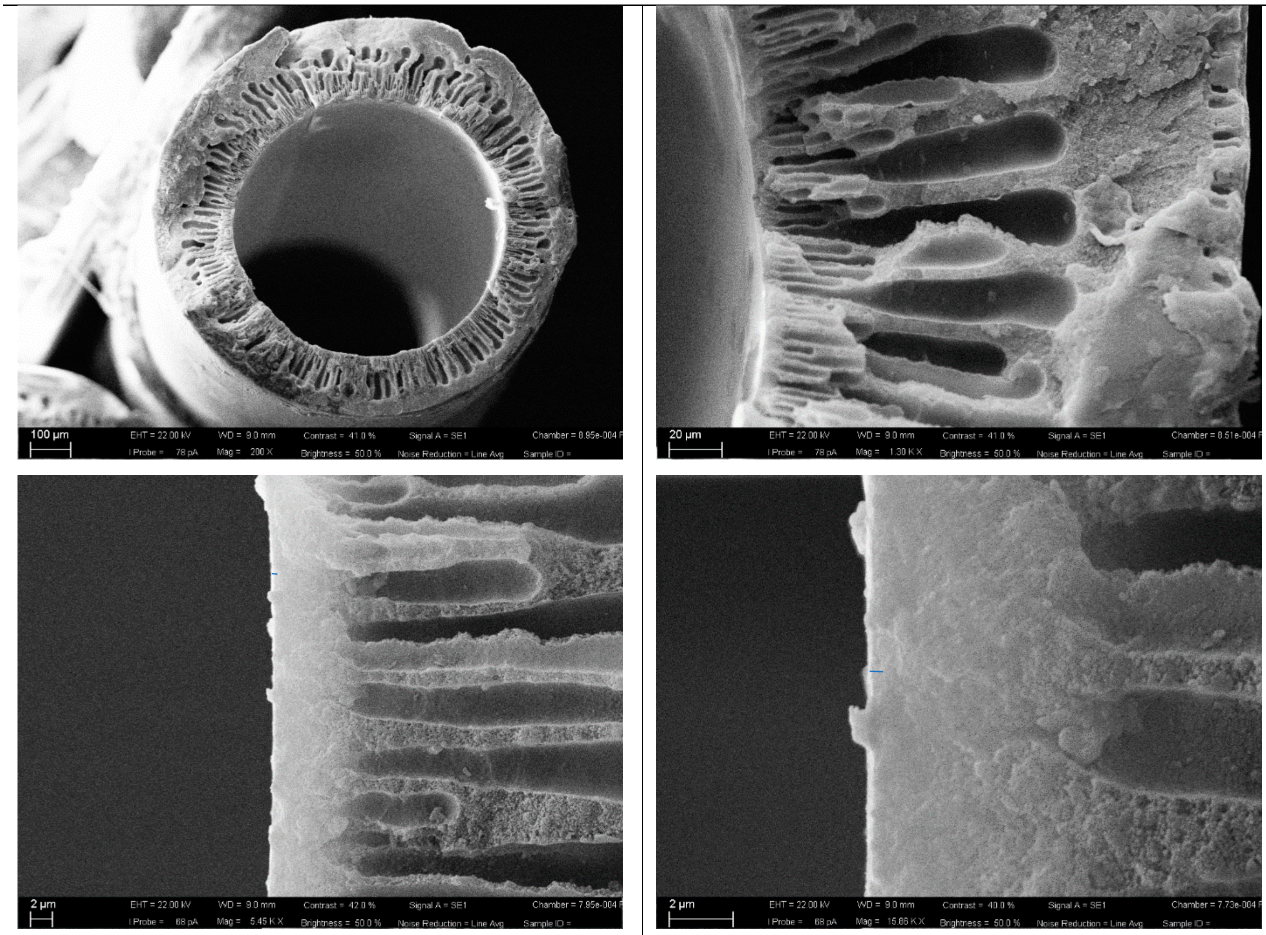

H2 (BF= NMP/water 20/80) | H3 (BF=NMP/water 30/70)

Figure S3. SEM images of H2 and H3 HFs: cross-section and magnification of the inner skin layer.
